# Neural substrates of neuropsychological profiles in dystrophynopathies: A pilot study of diffusion tractography imaging

**DOI:** 10.1371/journal.pone.0250420

**Published:** 2021-05-03

**Authors:** Laura Biagi, Sara Lenzi, Emilio Cipriano, Simona Fiori, Paolo Bosco, Paola Cristofani, Guia Astrea, Antonella Pini, Giovanni Cioni, Eugenio Mercuri, Michela Tosetti, Roberta Battini

**Affiliations:** 1 Laboratory of Medical Physics and Magnetic Resonance, IRCCS Fondazione Stella Maris, Calambrone, Pisa, Italy; 2 Department of Developmental Neuroscience, IRCCS Stella Maris, Calambrone, Pisa, Italy; 3 Department of Physics, University of Pisa, Pisa, Italy; 4 Department of Clinical and Experimental Medicine, University of Pisa, Pisa, Italy; 5 Pediatric Neurology Unit, Catholic University and Nemo Center, Policlinico Universitario Gemelli, Rome, Italy; University at Buffalo, UNITED STATES

## Abstract

**Introduction:**

Cognitive difficulties and neuropsychological alterations in Duchenne and Becker muscular dystrophy (DMD, BMD) boys are not yet sufficiently explored, although this topic could have a relevant impact, finding novel biomarkers of disease both at genetics and neuroimaging point of view. The current study aims to: 1) analyze the neuropsychological profile of a group of DMD and BMD boys without cognitive impairment with an assessment of their executive functions; 2) explore the structural connectivity in DMD, BMD, and age-matched controls focusing on cortico-subcortical tracts that connect frontal cortex, basal ganglia, and cerebellum via the thalamus; 3) explore possible correlations between altered structural connectivity and clinical neuropsychological measures.

**Materials and methods:**

This pilot study included 15 boys (5 DMD subjects, 5 BMD subjects, and 5 age-matched typically developing, TD). They were assessed using a neuropsychological assessment protocol including cognitive and executive functioning assessment and performed a 1.5T MRI brain exam including advance Diffusion Weighted Imaging (DWI) method for tractography. Structural connectivity measurements were extracted along three specific tracts: Cortico-Ponto-Cerebellar Tract (CPCT), Cerebellar-Thalamic Tract (CTT), and Superior Longitudinal Fasciculus (SLF). Cortical-Spinal Tract (CST) was selected for reference, as control tract.

**Results:**

Regarding intellectual functioning, a major impairment in executive functions compared to the general intellectual functioning was observed both for DMD (mean score = 86.20; SD = 11.54) and for BMD children (mean score = 88; SD = 3.67). Mean FA resulted tendentially always lower in DMD compared to both BMD and TD groups for all the examined tracts. The differences in FA were statistically significant for the right CTT (DMD vs BMD, p = 0.002, and DMD vs TD, p = 0.0015) and the right CPCT (DMD vs TD, p = 0.008). Concerning DMD, significant correlations emerged between FA-R-CTT and intellectual quotients (FIQ, p = 0.044; ρs = 0.821), and executive functions (Denomination Total, p = 0.044, ρs = 0.821; Inhibition Total, p = 0.019, ρs = 0.900). BMD showed a significant correlation between FA-R-CPCT and working memory index (p = 0.007; ρs = 0.949).

**Discussion and conclusion:**

In this pilot study, despite the limitation of sample size, the findings support the hypothesis of the involvement of a cerebellar-thalamo-cortical loop for the neuropsychological profile of DMD, as the CTT and the CPCT are involved in the network and the related brain structures are known to be implied in executive functions. Our results suggest that altered WM connectivity and reduced fibre organization in cerebellar tracts, probably due to the lack of dystrophin in the brain, may render less efficient some neuropsychological functions in children affected by dystrophinopathies. The wider multicentric study could help to better establish the role of cerebellar connectivity in neuropsychological profile for dystrophinopathies, identifying possible novel diagnostic and prognostic biomarkers.

## Introduction

Dystrophinopathies are the most common single gene disorders leading to muscle wasting, due to mutations in *DMD* gene. In-frame mutations generally produce abnormal but partly functional gene product *dystrophin*, which is part of a protein complex located in the muscle cell membrane, and are associated with Becker muscular dystrophy (BMD). The variable expression of the protein reflects the widely variable clinical phenotype observed in BMD children, in terms of motor and cardiorespiratory outcome [1]. On the contrary, mutations that disrupt the reading frame commonly result in the lack of dystrophin, leading to the most severe disease Duchenne muscular dystrophy (DMD) [2].

In addition to the well known peripheral muscular involvement, several studies have also reported a central nervous system involvement, resulting in cognitive difficulties and neuropsychological alterations in DMD boys [3–13], while less is known about possible neuropsychological impairments in BMD [14–16].

From a neuropathological point of view, the cognitive and neuropsychological involvement in DMD may be due to the lack of specific dystrophin isoforms in the brain. Two alternative full-length isoforms (Dp427B and Dp427P) are indeed expressed in the cerebral neocortex and two carboxy-terminal dystrophin proteins, Dp71 and Dp140, in the Purkinje cells of the cerebellar cortex [17–20]. Dp140 is expressed mainly in fetal tissue and in low quantity in adult brain and probably plays a role in the regulation of neuroglial specific gene expression. Dp71 expression gradually increases from the embryo until adult stage, becoming the major product of dystrophin in the brain, particularly in the hippocampus and in some layers of the cerebral cortex. The Dp71 function remains unknown but a role in the formation and/or stabilization of the dystrophin- associated complex and in glutaminergic synaptic maturation and function is supported by studies [21, 22].

In view of the localization of dystrophin isoforms in normal brain and of the neurocognitive involvement of DMD subjects, a possible role of cerebellum and of a complex cerebro-cerebellar network has been hypothesized in DMD [23].

Recently, an impairment of multitasking, problem solving, inhibition, working memory [24] and an implicit learning deficit [25] emerged in a group of 40 DMD boys without intellectual disability during school age. To examine this a selected DMD sample has allowed a targeted neurocognitive detection free from potential bias. Tasks requiring executive functions that have been explored in these studies are believed to activate a cortico-subcortical circuits which connect the prefrontal cortex, the basal ganglia and the cerebellum via the thalamus [26], thus strengthening the hypothesis of a possible involvement of the cerebellum as part of a more general involvement of the cerebellar-thalamo-cortical network.

Neuroimaging studies, although few in number, also corroborate the hypothesis of the involvement of cerebro-cerebellar loops in DMD: for example, positron emission topography (PET) analysis demonstrated in DMD patients glucose hypometabolism in brain areas that are typically rich in dystrophin (as cerebral cortex and cerebellum) [27, 28]. Moreover, the application of Magnetic Resonance Spectroscopy (MRS) in DMD patients revealed alterations in choline levels in both cerebellar white matter among others [29, 30]. A brain involvement in DMD has been also supported by functional MR studies that showed a reduced local synchronization of spontaneous activity of the neural networks in the motor cortex in patients with DMD [31] and a reduction of cerebral perfusion in DMD, regardless of the reduced grey matter volume [32].

Despite growing evidences of a brain dysfunction in DMD involving cerebellar networks for executive functions, to date few studies have explored white matter connectivity by Diffusion Weighted Imaging (DWI).

Brain tractography has been widely applied in developmental age, both in neurodevelopmental disorders, as ADHD [33] and autism spectrum disorders [34–36], and in neurological disorders, as leukodystrophies, cerebral palsy or cerebellar diseases [37–42].

Examples exist of the possible double involvement of brain and muscle in neuromuscular diseases, demonstrated as white matter abnormalities by DWI tractography. In particular, alterations of white matter projection, association and commissural fibres have been described in myotonic dystrophy type 1 [43–45] and alterations of diffusion coefficient of white matter have been revealed for merosin-deficient congenital muscular dystrophy [46, 47].

In DMD patients, some evidence of microstructural differences in scalar measures (Fractional Anisotropy, FA, and Mean Diffusivity, MD) has been shown in the occipital regions [48] and in the splenium of corpus callosum, with a correlation with intellectual quotients [49]. Alterations in diffusion in the prefrontal cortex and hippocampus emerged also in *mdx* mice, the animal model of DMD [50].

The purposes of this explorative study are threefold:

1. to analyze the neuropsychological profile of a group of DMD and BMD boys without cognitive impairment with an assessment of their executive functions. Because of the variable expression of the protein in BMD, we might speculate that the BMD neuropsychological phenotype could be different compared to DMD;
2. to explore the structural connectivity in DMD, BMD and age-matched controls focusing on cortico-subcortical tracts which connect frontal cortex, basal ganglia and cerebellum via the thalamus. Mean FA values along each of the selected tracts were calculated, used as a measure of altered structural connectivity and compared among groups. We hypothesized that DMD boys had lower FA in the tracts compared to BMD and controls;
3. to explore possible correlations between altered FA values and clinical measures of neuropsychological function in DMD and BMD groups, even if in a pilot study, in order to identify possible functional neuroimaging biomarkers of the neuropsychological profile for further investigations on wider sample. We hypothesized that reduced FA corresponded to higher functional impairment.

## Materials and methods

### Subjects

This study involved patients enrolled by IRCCS Stella Maris Foundation, University of Pisa, one of the Centers members of DMD Italian Network. In total, 15 subjects participated in the study: 5 DMD subjects, 5 BMD subjects and 5 age-matched typically developing (TD) boys.

Different reasons made recruiting difficult in DMD children: a) the rarity of the DMD disease; b) the difficulty for the patients families to move from long distances; c) the participation of many DMD patients in experimental trials which already involve several clinical monitoring; d) the refusal of patients to participate in a study involving a brain Magnetic Resonance Imaging (MRI) protocol which is not part of the routine follow up. Therefore, only 5 DMD boys (mean age: 10.1 years old; range: 7–13 years) were enrolled in the study, two coming from Bologna.

Because of the involvement of dystrophin in all dystrophinopathies, 5 BMD boys (mean age: 13.1 years old; range: 11–15 years) were recruited.

The inclusion criteria for the study subjects were the following: i) DMD and BMD boys with proven mutation in the dystrophin gene; ii) the availability of a neuropsychological evaluation assessing cognitive and neuropsychological functioning, according to the protocol already discussed [24]; iii) no cognitive impairment (IQ<70) or any associated neuropsychiatric disorders (drug-resistant epilepsy, autism spectrum, attention deficit and hyperactivity) or any additional neurosensory deficits; iv) steroid treatment and/or other experimental drug stable for at least six months.

Moreover, 5 age-matched typically developing (TD) boys (mean age: 9.5 years old; range: 7–12 years) were enrolled among the children performing brain MRI for headache without other neurological signs and with normal brain MRI.

All the children were recruited only if they have not MRI contraindications and if they were be able to collaborate to MRI exam, lying supine in the scanner for at least 30/40 minutes.

The study was approved by Tuscany Pediatric Ethics Committee and a specific informed consent form has been signed by all parents and subjects included in the study.

### Methods

#### Neuropsychological assessment

Both the DMD and the BMD children were assessed using a neuropsychological assessment protocol including cognitive and executive functioning assessment, a simplified format compared to the one previously published [24]. Concerning cognitive evaluation, we performed Wechsler Intelligence Scale for children (WISC-IV) for Full Intellectual Quotients (FIQ) and Working Memory Index (WMI) for all the dystrophynopathy-subjects [51]. With regard to executive functions, Inhibition test of NEPSY-II [52] and Tower Of London (TOL) tests [53] were administered.

#### MRI acquisition

MRI data were acquired by using a 1.5T MRI scanner (1.5T GE HDx) at MRI Laboratory of IRCCS Stella Maris Foundation. The acquisition protocol consisted of: (1) Isotropic high-resolution T1-weighted sequence (3D BRAVO) with slice thickness = 1 mm, Field of view (FOV) = 256 mm X 256 mm, matrix = 256 X 256; Time of repetition (TR)/ Time of echo (TE) = 450/5.18 ms, flip angle (fa) = 13°; (2) isotropic diffusion weighted sequence using a 2D single-shot EPI; including 30 non-collinear encoding directions with b value of 1000 and one additional volume without diffusion gradients (b0), slice thickness = 3 mm; FOV = 240 mm X 240 mm; matrix = 80 x 80; TR/TE = 13000/115.8 ms.

#### MRI analysis

Brain tissue segmentation was performed using FreeSurfer based on 3D T1-weighted images as in [54]. FreeSurfer is used as pre-processing workflow for structural MRI data to perform volumetric segmentation and cortical reconstruction through 31 processing steps [55]. The processing steps included skull stripping, motion correction, removal of non-brain tissue, spherical surface registration, tissue segmentation and parcellation of the cortex into anatomical regions, obtaining white matter (WM), gray matter (GM), cerebrospinal fluid (CSF) and subcortical gray matter structures for each subject. FreeSurfer provides several descriptive features of mentioned structures as well, such as volume that was considered in our analysis.

DWI data were preprocessed to correct image artifacts caused by involuntary head motion, cardiac pulsation, and intensity inhomogeneities by using FSL tools (https://fsl.fmrib.ox.ac.uk/fsl). After preprocessing, a color-encoded track-density image was generated to support the identification of Regions of Interest (ROI) for tract reconstruction. Fiber tractography (MRtrix package) was performed using constrained spherical deconvolution (CSD) with a maximum number of streamlines of ten thousand, by using the iFOD2 algorithm that facilitates more accurate fiber reconstruction in heavily curved regions [56]. Anatomically-Constrained Tractography (ACT) option was applied by using the 5-types-tissue (5TT) segmented images to correct tractography and to increase anatomical plausibility of the reconstructed fibers based on prior information [57], discarding streamlines that are anatomically unfeasible.

Three tracts of interest were identified considering cortico-subcortical tracts, which connect frontal cortex, basal ganglia and cerebellum via the thalamus. In particular, Cortico-Ponto-Cerebellar tract (CPCT), Cerebellar-Thalamic Tract (CTT) and Superior Longitudinal Fasciculus (SLF) were selected in each hemisphere of all subjects. Cortico-Spinal Tract (CST) was identified as “control” tract, since it is not expected to be associated with the cognitive measure of interest. Mean FA value along the tracts were extracted.

A single rater, blinded to the participant status (DMD/BMD/TD), performed tractography, drawing manually appropriate seeding and inclusion ROIs for each tract.

Tracts were checked by two experienced raters on all subjects to verify trajectory and anatomic landmarks described in atlases of human WM and to check false-positive streamlines within the pathways.

The CPCT of left hemisphere was selected setting a seeding ROI in the right middle cerebellar peduncle and an inclusion ROI in the left posterior limb of the internal capsule, as previously [41]. An add inclusion ROI was positioned in a frontal WM area of left hemisphere [58, 59]. The CPCT of right hemisphere was obtained using the same procedure, selecting manually the homologous ROIs of the contralateral hemispheres.

To define the left CTT, a seed in the left superior cerebellar peduncle and a spherical inclusion ROI on the left thalamic WM were chosen [41]. For the right CCT, homologue ROIs were selected in the contralateral hemispheres.

As in Kamali et al. [59] to identify SLF, and for each hemisphere, the first ROI was placed over the green association bundles just superolateral to the cingulum on the color-encoded track-density at the most posterior part of the corpus callosum. The second ROI was placed over the fibers generated on the superolateral aspect of the cingulum at the coronal plane passing though the mid thalamus.

For the delineation of left CST, a spherical seeding ROI was placed in the left white matter in correspondence of the precentral gyrus, at the level of hand omega. An inclusion ROI was selected between the transverse pontine fibers and the middle cerebellar peduncle [60]. The CST of right hemisphere was obtained, drawing the homologous ROIs in the contralateral hemispheres.

The number of streamlines and FA was calculated for each tract examined; FA is an invariant scalar measure that describes the degree of diffusion anisotropy, reflecting fibre density, axonal diameter and myelination, and varies between 0 (equal diffusion in all directions) and 1 (highly directional diffusion).

To improve the specificity of quantitative DTI metrics (i.e. FA), and to take in account Partial Volume Effects (PVE), we calculate the tract volume, to use it as a covariate not-of-interest [61]. In fact, Vos et al. (2011) have shown that tract volume, orientation, and curvature are PVE-modulating factors that can affect the estimation of diffusion metrics when sampled along the tract. The tract volume, in particular, contributes to the explanation of the observed differences in DTI measures between populations. The volume of the tracts was obtained by: i) generating a binary mask starting from the tract; ii) calculating the number of voxels contained within the mask; iii) converting the number of voxels in mm^3^, multiplying it by the voxel volume (image resolution).

#### Statistical analysis

For each subject, a complete set of neuropsychological clinical measures was included in the analyses. The mean FIQ and WMI were considered in the normal range according to the Diagnostic Mental Index if the mean value was 100 and the SD was 15. The Inhibition test of NEPSY-II was measured as mean 10 and SD 3. TOL results were expressed with T Score (mean = 50; SD = 10).

For all subjects, the number of streamlines and FA were calculated for each reconstructed tract (CTT, CPCT, SLF and CST). Within each subject, a paired sample t test was used to compare mean FA and number of streamlines between the right and left side of each tract.

A general linear model was used to determine the difference among groups for FA and clinical measures, and post hoc pair-wise comparisons were performed. Effect size was calculated for significant differences, by using the Hedges’s g parameter, that corrects the standard Cohen’s d effect size for potential bias due to small samples [62].

For tracts that showed altered connectivity among groups, ANCOVA analyses were performed using age and the tract volume as covariate. Moreover, for these tracts, the relationship with clinical measures was explored.

Statistical analyses were performed by using SPSS, Version 2.0 (IBM, Armonk, New York).

To correct for multiple comparisons, we used the method of Benjamini and Hochberg [63], which recalculates the level of significance in terms of p-values, limiting the false discovery rate (FDR) to 5%.

## Results

### Neuropsychological profile in DMD and BMD children

The neuropsychological profile of the small group of BMD children resulted almost comparable to that already described in DMD [24]. In fact, regarding intellectual functioning, a major impairment in WMI compared to the general intellectual functioning was observed both for DMD (mean score = 86.20; SD = 11.54) and for BMD children (mean score = 88; SD = 3.67). Worse performances were confirmed in the Switching task of the Inhibition test (NEPSY-II) (mean score for DMD boys = 5.60; SD = 2.70; mean score for BMD boys = 6; SD = 1.87) compared to the other tasks of that test. In TOL test, the BMD boys showed worse impairments in “total correct answers” than DMD (mean score for DMD = 45.40; SD = 11.01; mean score for BMD = 36.60; SD = 13.35).

No significant difference emerged between the BMD and DMD groups enrolled in the study in neuropsychological measures, except for the Denomination task of the Inhibition test (NEPSY-II), where BMD obtained worse performances (p = 0.014).

Table 1 shows mean scores and SDs of cognitive and executive functioning tests in BDM and DMD groups.

**Table 1:** Intellectual and executive functioning in DMD and BMD cohorts.

	DMD	BMD
	5 subjects	5 subjects
	*Mean (SD)*	*Mean (SD)*
***WISC-IV Index***		
Working Memory Index	86.2 (11.5)	88 (3.7)
Full Intellectual Quotient	100.2 (7.0)	95.2 (14.2)
***Inhibition test (NEPSY-II) (standard score)***		
Denomination Total	7.2 (1.6)*	6.4 (0.6)*
Inhibition Total	7.6 (2.1)	7.4 (1.3)
Switching Total	5.6 (2.7)	6.0 (1.9)
***TOL test (T score)***		
Total corrected	45.4 (11.0)	36.6 (13.4)

* = statistically significant differences

### Fiber tracts reconstruction

**Fig 1:**
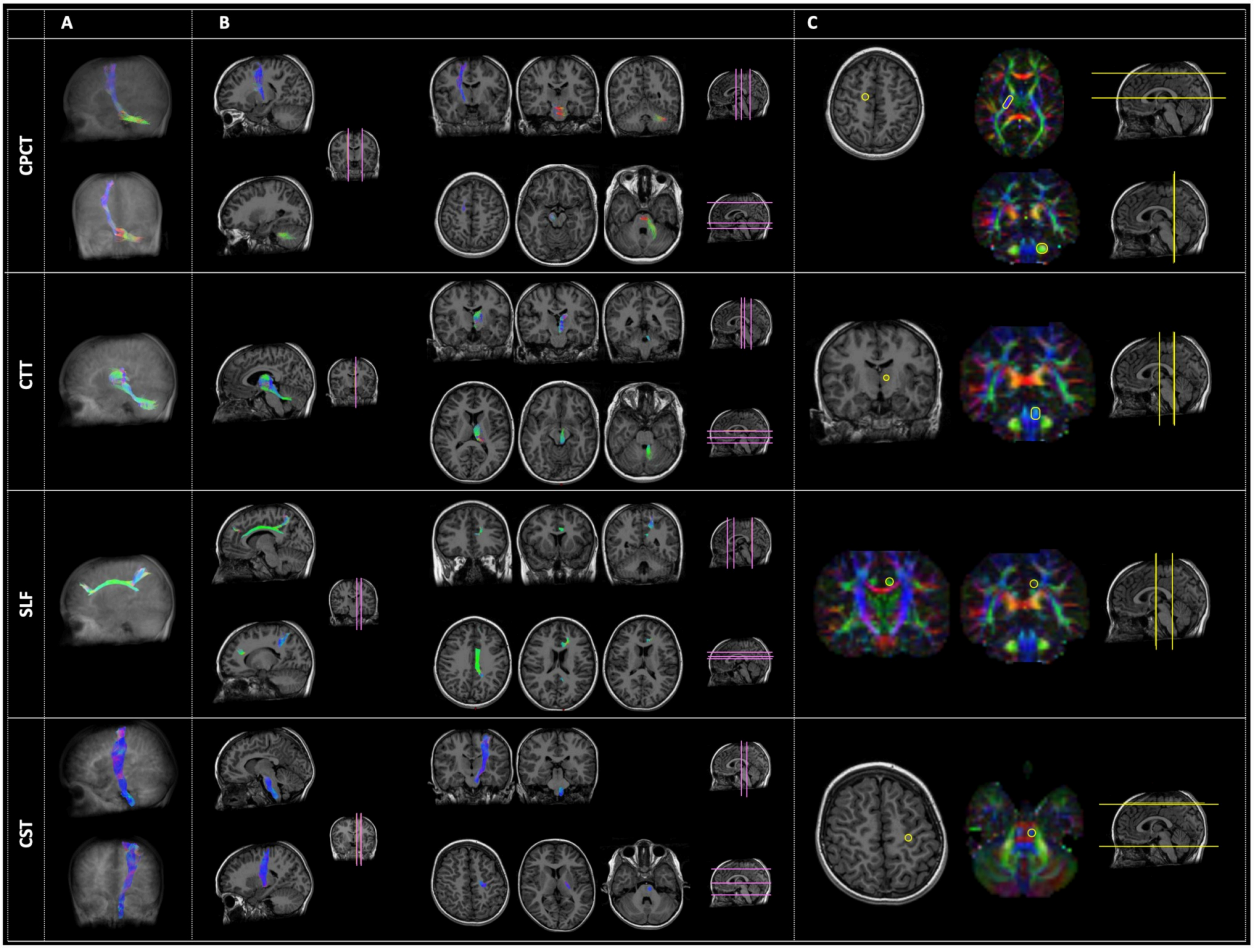
Examples of the four tracts of interest: From the top, the Cortico-Ponto-Cerebellar Tract (CPCT), the Cerebellar-Thalamic Tract (CTT), the Superior Longitudinal Fasciculus (SLF) and the Cortico-Spinal Tract (CST). Panel A represents the three-dimensional reconstruction of each tract in a glass brain. In panel B, the pathway of tractography is depicted in some representative slices with different orientation: Sagittal (right side of the panel), coronal (left side of the panel, upper rows), and axial (left side of the panel, lower rows). The location of these slices is reported in pink color on a reference brain. Panel C reports the ROIs (yellow circles) used for the tractography of each tract, both in anatomical images or in the diffusion-encoded-color (DEC) maps. Analogously to panel B, the location of these images is represented in yellow color on a reference brain. The order of representation of images corresponds to the Right-Left (R-L) direction for sagittal images, to the Anterior-Posterior (A-P) direction for coronal images, and to the Superior-Inferior (S-I) direction for axial ones. The color code of tractography pathways and DEC maps is the standard red-green-blue (RGB) code for diffusion: red for R-L direction, blue for S-I, and green for A-P.

All fiber tracts were successfully extracted on each hemisphere of each subject (Fig 1).

The number of streamlines was >10 in each of the examined tracts. No statistically significant differences for number of streamlines of all the examined tracts were found between the left and the right side within each group of children (complete dataset in S1 Table), neither between the three groups of subjects.

Moreover, within each group of subjects, no significant differences are found between the mean FA of the right and the left representations of all examined tracts.

Mean FA resulted tendentially always lower in DMD compared to both BMD and TD groups for all the examined tracts, with the exception of the right SLF (FA-R-SLF), which showed the same values of FA in DMD and TD.

A statistically significant difference emerged between DMD and BMD in the mean FA value of the right CTT (FA-R-CTT) (p = 0.002), with a “huge” effect size (g = 3.3). Moreover, a statistically significant difference emerged between DMD and TD in the mean FA-R-CTT (p = 0.0015) and in the FA-R-CPCT (p = 0.008). The effect size was “huge” in both cases (g = 2.8 for FA-R-CTT and g = 1.8 for FA-R-CPCT).

No statistically significant differences emerged among groups for FA values of bilateral SLF and bilateral CST. The results are shown in Table 2.

**Table 2:** Mean FA in all the examined tracts in DMD, BMD and TD.

	DMD	BMD	TD
	*Mean (SD)*	*Mean (SD)*	*Mean (SD)*
**FA-L-CTT**	0.34 (0.02)	0.36 (0.03)	0.36 (0.01)
**FA-R-CTT**	0.32 (0.01)*	0.36 (0.01)*	0.37 (0.02)*
**FA-L-CPCT**	0.48 (0.01)	0.49 (0.02)	0.49 (0.03)
**FA-R-CPCT**	0.46 (0.02)*	0.49 (0.03)	0.49 (0.01)*
**FA-L-SLF**	0.37 (0.04)	0.41 (0.07)	0.39 (0.05)
**FA-R-SLF**	0.36 (0.01)	0.38 (0.04)	0.36 (0.03)
**FR-L-CST**	0.49 (0.03)	0.51 (0.03)	0.50 (0.03)
**FA-R-CST**	0.49 (0.04)	0.50 (0.03)	0.49 (0.02)

FA-L-CTT: FA of the left Cerebellar-Thalamic Tract; FA-R-CTT: FA of the right Cerebellar-Thalamic Tract; FA-L-CPCT: FA of the left Cortico-Ponto-Cerebellar Tract; FA-R-CPCT: FA of the right Cortico-Ponto-Cerebellar Tract; FA-L-SLF: FA of the left Superior Longitudinal Fasciculus; FA-R-SLF: FA of the right Superior Longitudinal Fasciculus; FA-L-CST: FA of the left Cortico-Spinal Tract; FA-R-CST: FA of the right Cortico-Spinal Tract. DMD: Duchenne muscular dystrophy children; BMD: Becker muscular dystrophy children; TD: typical developing children.

* = statistically significant differences with DMD.

Adding age and tract volume as covariates in the statistical analyses, the significances remain valid, according to S2 Table.

**Fig 2:**
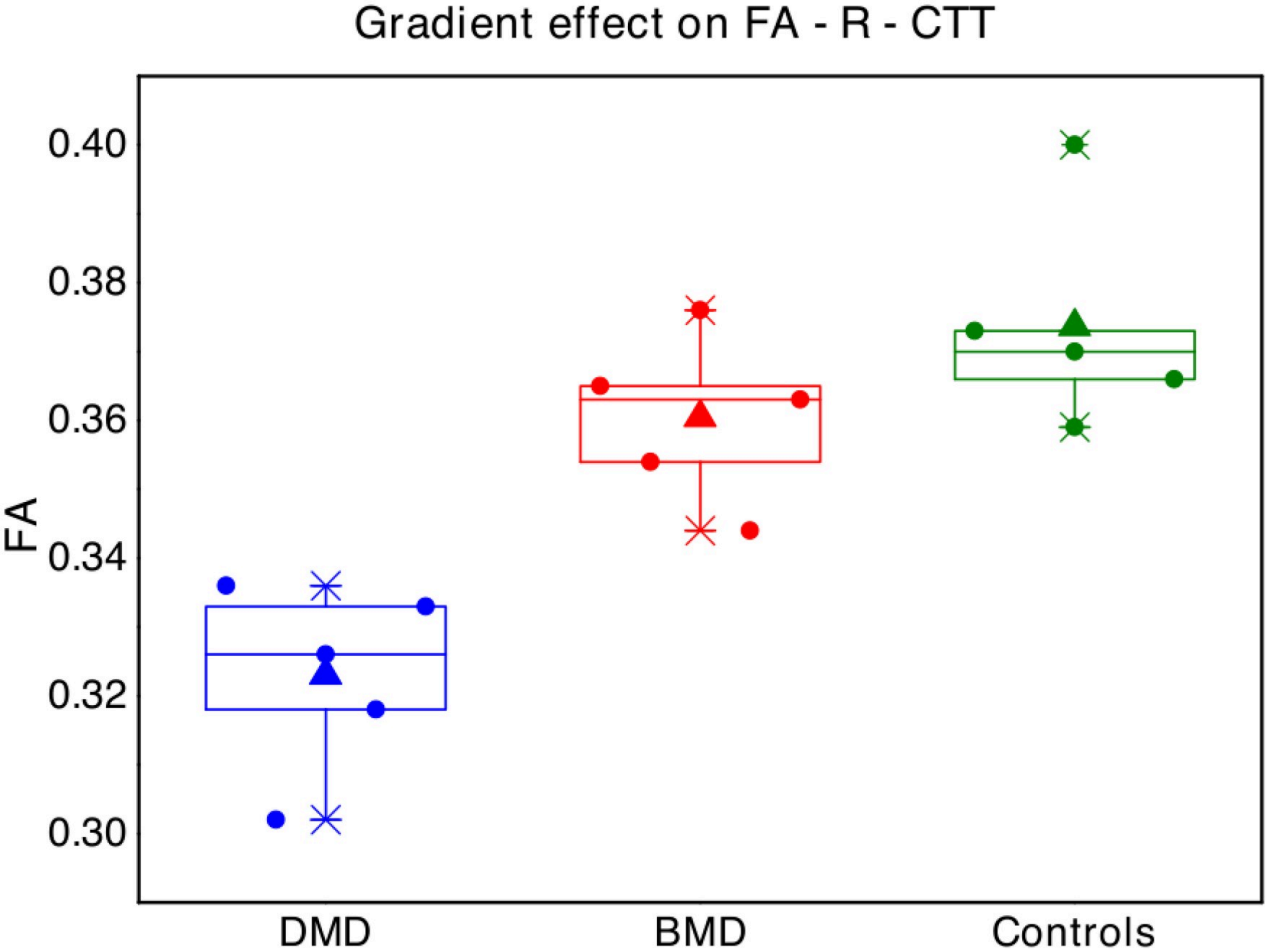
Boxplot represent FA values in the right cerebellar-thalamic tract for each single subject of the three groups (blue points = Duchenne muscular dystrophy children -DMD; red = Becker muscular dystrophy children -BMD; green = typical developing children -TD). The box represents the interval between the 25° and 75° percentile of data, the lines correspond to the median values, the triangles to the mean values.

Interestingly, with regard to FA-R-CTT, a possibly “gradient effect” was observed, with mean FA of BMD children resulting in the middle between mean FA of DMD and mean FA of controls (Fig 2).

### Correlations between FA fiber tracts and neuropsychological profile

**Fig 3:**
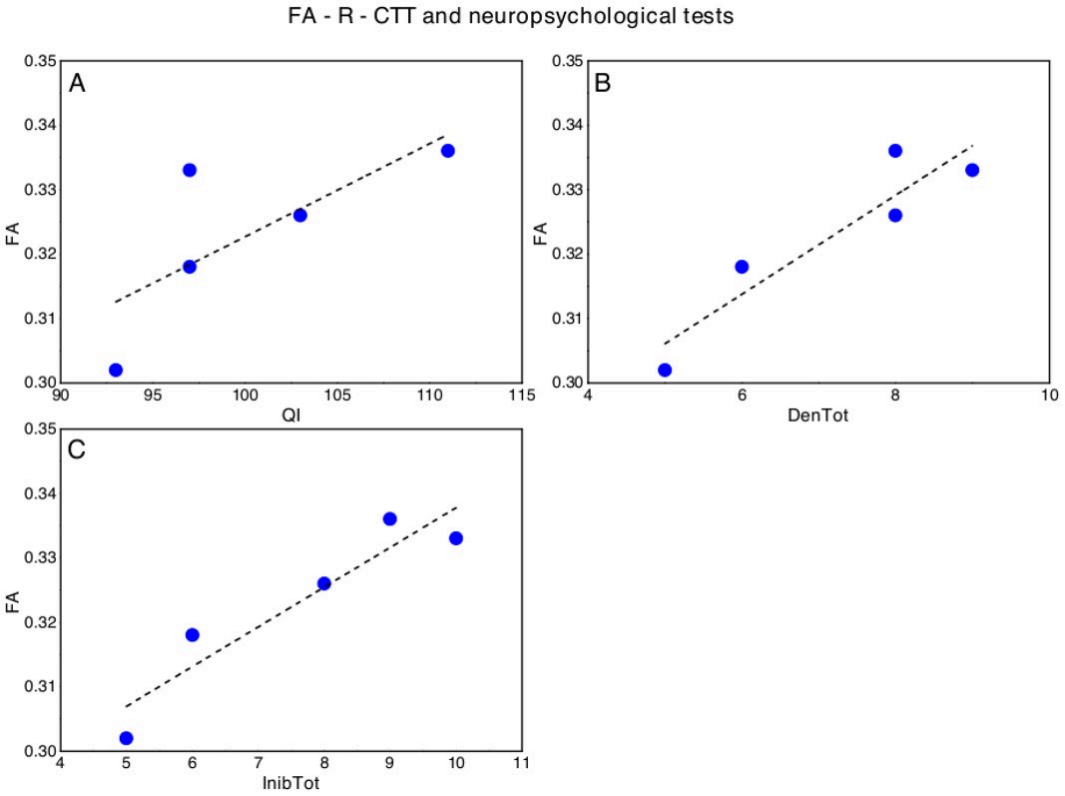
Significant correlations between FA-R-CTT and neuropsychological tests in DMD, in particular with respect to (A) Full Intellectual Quotient (FIQ, WISC-IV), (B) Denomination Total (DenTot, Inhibition test- NEPSY II) and (C) Inhibition Total (InhibTot, Inhibition test- NEPSY II). The dotted lines represent the linear fit of the data with a function y = a + b*x. A) FA-R-CCT versus FQI: a = 0.18±0.08, b = 0.001±0.0008, Pearson’s R = 0.74, Adj R2 = 0.40; B) FA-R-CCT versus: a = DenTot: 0.27±0.01, b = 0.008±0.0002, Pearson’s R = 0.93, Adj R2 = 0.81; C) FA-R-CCT versus InibTot: a = 0.28±0.01, b = 0.006±0.0001, Pearson’s R = 0.94, Adj R2 = 0.84.

Concerning DMD, significant correlations emerged between FA-R-CTT and FIQ (p = 0.044; ρ_s_ = 0.821), Denomination Total (p = 0.044; ρ_s_ = 0.821) and Inhibition Total (p = 0.019; ρ_s_ = 0.900) of the Inhibition test (NEPSY-II) (Fig 3). No other significant correlation emerged between FA-R-CTT and neuropsychological tests.

**Fig 4:**
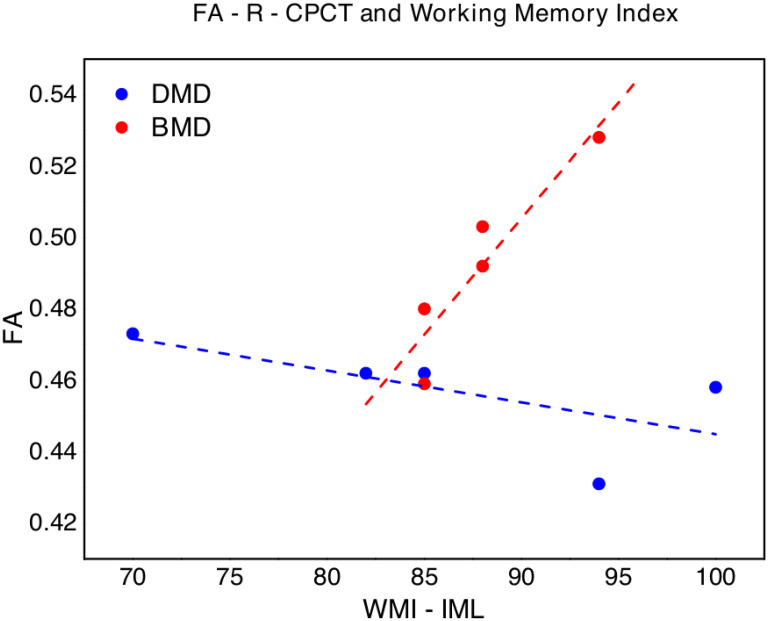
FA of the right corticopontocerebellar tract (FA-R-CPCT) values versus Working Memory Index (WMI, WISC-IV) in DMD (blue points) and BMD (red points). The dotted lines (blue for DMD and red for BMD respectively) represent the linear fit of the data with a function y = a + b*x. DMD: a = 0.53±0.05, b = -0.001±0.0005, Pearson’s R = -0.66, Adj R^2^ = 0.24; BMD: -0.08±0.13, b = 0.007±0.0001, Pearson’s R = 0.93, Adj R^2^ = 0.81.

We also found a slight negative correlation between FA-R-CPCT and WMI (p = 0.027; ρ_s_ = -0.872), while, regarding BMD, a significant correlation emerged between FA-R-CPCT and WMI (p = 0.007; ρ_s_ = 0.949) (Fig 4). No other significant correlation emerged between FA-R-CPCT nor FA-R-CTT and neuropsychological tests in BMD.

## Discussion

In this study, we explored the neuropsychological profile in a group of DMD and BMD boys without intellectual impairment and we investigate possible correlations with the obtained DWI data comparing DMD, BMD and controls. We explored WM microstructure in tracts that are known or supposed to be part of executive functions networks by investigating FA, as the measure of disrupted connectivity. In particular, we explored two cerebellar tracts, separately on the right and left sides: the CTT, which is the main efferent pathway from cerebellum, and the CPCT, which is the major input of the cerebellum from the cerebral cortex, focusing specifically on the fibers originating from the frontal area. Moreover, the SLF, involved in fronto-striatal connections and in executive functions, as already described in literature [64], has been included, separately on the right and left sides. The CST was included as a control tract not expected to be associated with cognitive functions.

Our neuropsychological results suggest that the BMD neuropsychological phenotype may be similar to DMD one, due to the involvement of specific dystrophin isoforms in the brain. The variable expression of the dystrophin in BMD can account for the variability of neurocognitive profile. Our BMD sample showed a major impairment of working memory compared to the general intellectual functioning and a failure in executive functions like problem solving, inhibition and shifting abilities, as already described in literature for DMD [7, 11, 24].

Regarding DWI data, the overall mean FA resulted lower in DMD children than in the other groups of subjects for the examined tracts, suggesting a possible alteration in WM microstructural integrity. More specifically, the cerebellar connectivity seemed to be more compromised, compared to SLF and CST in the DMD group.

Even if with few and explorative contributions, possible altered WM connectivity has been already described in DMD boys [48, 49]. We suppose that the lack of cerebral dystrophin protein in DMD children, already during neural development, may be responsible for reduced fiber coherence, and altered myelination and axonal density in the explored tracts. This speculation may be supported also by the results obtained in BMD children, that variably express dystrophin protein, who showed an intermediate mean FA in some of the examined tracts, with a sort of “gradient effect” between DMD and controls, at least in tracts that might be responsible for specific cerebral symptoms (i.e. neuropsychological dysfunction). A greater effect regarding differences between DMD and BMD groups seems to be observed in CTT than in CPCT, but the results must be confirmed in wider samples, considering also the slight difference in age between the two groups.

In fact, some studies have shown a development and maturation of executive functioning, using neuropsychological or neuroimaging approaches, both in normotypical children and adolescents [65–67] and in clinical populations, such as children with autism spectrum disorders and ADHD, in which emerged that not until 7 to 9 years of age switch flexibility begin operating [68, 69].

Our recent results about neuropsychological profile confirmed a similar neuropsychological maturation also in DMD [70].

In order to explore possible functional correlates of altered connectivity for specific tracts, we thus studied the relationship between mean FA values in the cerebellar tracts that showed reduced FA and neuropsychological measures. Indeed, we demonstrated a less widespread involvement in BMD compared to DMD. In detail, regarding DMD children, we observed significantly lower FA-R-CTT in boys with lower FIQ and a major impairment in inhibition abilities.

FA-R-CPCT seems to show a different behavior with respect to the working memory abilities for DMD and BMD boys. This result may be due to the small sample size and must be verified in a next wider analysis, but could also underline unexpected mechanisms of maladaptive neural plasticity for DMD, which may produce abilities globally slightly reduced in DMD compared to BMD children.

Overall, these findings support the hypothesis of the involvement of a cerebellar-thalamo-cortical loop for the neuropsychological profile of DMD, as the CTT and the CPCT are involved in the network and the related brain structures are known to be implied in executive functions.

It has long been known that frontal cortex, in particular prefrontal area, plays a central role in global aspect of general intelligence [71] and executive functions [69] across the lifespan; thanks to connections between the prefrontal cortex and other brain regions, the neural substrates of executive functions include also the parietal cortex, the anterior cingulate cortex, and subcortical regions as the striatum and the cerebellum [72]. Moreover, the superior cerebellar peduncle, involved in CTT, and the posterior limb of internal capsule, involved in CPCT, are thought to be key components of the circuit [73]. Recent data have also emphasized a role of the thalamus, in particular mediodorsal nucleus, in cognition and executive functions because of its significant interconnectivity with prefrontal cortex [74, 75].

More in detail, a bilateral contribution of cerebellum in DMD seem to be suggested by our findings. In fact, both CTT which originates from the right cerebellum and CPCT fibers which, originating from the right frontal cortex, project to the pontine nuclei and cross the midline, thus terminating in the contralateral half of cerebellum [76], resulted more damaged in DMD than in BMD and controls. In literature, a bilateral contribution of cerebellum in executive functions is reported. For example, a cross cerebral-cerebellar circuitry with left prefrontal cortex predominantly involved and strong right cerebellum activation has been shown for verbal working memory [77]. However, a fMRI study has demonstrated a bilateral cerebellar activation for working memory paradigms, while other executive function tasks showed converging activation in lobules VI, Crus I and left VIIB of cerebellum [78]. Moreover, left and right cerebellum involvement in switching attention has been demonstrated [79]. To verify if differences in FA values were related to smaller brain size of DMD, we performed post hoc ANCOVA analyses to compare brain size between groups, using age as covariate. We found that DMD has significant smaller volumes of the cerebellar Gray Matter (Cereb-GM) both at a global level (p = 0.006), both for each hemisphere separately (L Cereb-GM p = 0.006, R Cereb-GM p = 0.007), than TD, confirming the involvement of cerebellum in the distrophynopathies.

The great limitation of the study is due to the small sample size that limits the possibility to correlate the neuroimaging data with the neuropsychological findings in a robust manner and suggest caveats in the interpretation of results. The enlargement of the sample could help us to better establish the role of cerebellar connectivity in neuropsychological profile for dystrophinopathies.

The slight unexpected negative correlation between FA-R-CPCT and WMI in DMD children needs to be further explored, also in consideration of the general difficulty of tracking CPCT, that is a long tract that sharply turns between cerebellum and cerebrum, and consequently of calculating the FA along it. This is evident also in the high values of standard deviation for the number of streamlines of CPCT, in particular in DMD, suggesting higher variability in fiber tracking among subjects within the same group.

Moreover, to increase the robustness and reliability of the data, it could be useful to replicate the study with state-of-art DWI acquisition techniques. In particular, 3T MR scanners, with new generation gradients, could allow acquisitions at higher spatial resolutions, in a multi-shell approach, in reasonable times of acquisition, allowing to implement multiple compartment models of diffusion, maximizing the accuracy of diffusion measurements and reducing PVE contributions.

Furthermore, DMD and BMD children enrolled in the study underwent only a brief neuropsychological assessment, and a more detailed evaluation could help not only to better describe the role of WM abnormalities but also, for BMD children, to define their neuropsychological profile.

In conclusion, to our knowledge, this is the first study to specifically explore the role of the cerebellar-thalamo-cortical network in DMD boys using DWI-based connectivity measures. Our results suggest that altered WM connectivity and reduced fibre organization in cerebellar tracts, probably due to the lack of dystrophin in the brain, may render less efficient some neuropsychological functions in children affected by dystrophinopathies. The findings lead us to identify possible functional neuroimaging biomarkers for the neuropsychological profile of DMD without intellectual disability.

## Supporting information

S1 Table Mean number of streamlines in the examined tracts in DMD, BMD and TD. (DOCX)

S2 Table Statistical significance (p-value) in the analyses without and with covariates. (DOCX)
