# Correction: Neural substrates of neuropsychological profiles in dystrophynopathies: A pilot study of diffusion tractography imaging

**DOI:** 10.1371/journal.pone.0314785

**Published:** 2024-11-26

**Authors:** Laura Biagi, Sara Lenzi, Emilio Cipriano, Simona Fiori, Paolo Bosco, Paola Cristofani, Guia Astrea, Antonella Pini, Giovanni Cioni, Eugenio Mercuri, Michela Tosetti, Roberta Battini

The affiliation for the eighth author is incorrect. The correct affiliation is not indicated. Antonella Pini, is not affiliated with #2 but with: Pediatric Neuromuscular Unit, IRCCS Istituto delle Scienze Neurologiche di Bologna.
