# Supplementary material for: Neural substrates of neuropsychological profiles in dystrophynopathies: A pilot study of diffusion tractography imaging
Source: PLoS One. 2021 May 3;16(5):e0250420. doi: 10.1371/journal.pone.0250420 (PMC8092766; doi:10.1371/journal.pone.0250420)
Supplement: S1 Table — (DOCX) [file pone.0250420.s001.docx]

**S1 Table. Mean number of streamlines in the examined tracts in DMD, BMD and TD**

|  | **nCTT**  *Mean (SD)* | | **nCPCT**  *Mean (SD)* | | **nSLF**  *Mean (SD)* | | **nCST**  *Mean (SD)* | |
| --- | --- | --- | --- | --- | --- | --- | --- | --- |
|  | **Left** | **Right** | **Left** | **Right** | **Left** | **Right** | **Left** | **Right** |
| **DMD** | 10000  (0) | 8527 (2937) | 1076 (1251) | 156  (213) | 10000  (0) | 10000  (0) | 9262 (575) | 9260 (264) |
| **BMD** | 6480  (4012) | 5162  (4524) | 798  (323) | 772  (768) | 10000  (0) | 10000  (0) | 9277 (446) | 9396 (600) |
| **TD** | 8390 (2266) | 9906  (210) | 1283  (815) | 417  (377) | 10000  (0) | 10000  (0) | 8882 (685) | 8967 (721) |

nCTT: number of streamlines in Cerebellar-Thalamic Tracts; nCPC: number of streamlines in Cortico-Ponto-Cerebellar Tracts; nSLF: number of streamlines in Superior Longitudinal Fasciculus; nCST: number of streamlines in Cortico-Spinal Tracts. DMD: Duchenne muscular dystrophy children; BMD: Becker muscular dystrophy children; TD: typical developing children. The SD equal to 0 means that all tracts of all subjects were selected finding the maximum number of allowed streamlines (10.000).
