# Supplementary material for: Neural substrates of neuropsychological profiles in dystrophynopathies: A pilot study of diffusion tractography imaging
Source: PLoS One. 2021 May 3;16(5):e0250420. doi: 10.1371/journal.pone.0250420 (PMC8092766; doi:10.1371/journal.pone.0250420)
Supplement: S2 Table — (DOCX) [file pone.0250420.s002.docx]

**S2 Table. Statistical significance (p-Value) in the analyses without and with covariates.**

|  |  | **Covariate** | | |
| --- | --- | --- | --- | --- |
|  |  | **None** | **Tract Volume** | **Age** |
| **DMD vs BMD** | **FA-R-CTT** | **0.002** | **0.004** | **0.011** |
| **DMD vs TD** | **FA-R-CTT** | **0.0015** | **0.003** | **0.002** |
|  | **FA-R-CPCT** | **0.008** | **0.012** | **0.006** |
